# Ectopic Expression of Arabidopsis Glycosyltransferase *UGT85A5* Enhances Salt Stress Tolerance in Tobacco

**DOI:** 10.1371/journal.pone.0059924

**Published:** 2013-03-22

**Authors:** Yan-Guo Sun, Bo Wang, Shang-Hui Jin, Xiao-Xia Qu, Yan-Jie Li, Bing-Kai Hou

**Affiliations:** The Key Lab of Plant Cell Engineering and Germplasm Innovation, Education Ministry of China; School of Life Science, Shandong University, Jinan, Shandong, P. R. China; Purdue University, United States of America

## Abstract

Abiotic stresses greatly influence plant growth and productivity. While glycosyltransferases are widely distributed in plant kingdom, their biological roles in response to abiotic stresses are largely unknown. In this study, a novel Arabidopsis glycosyltransferase gene *UGT85A5* was identified as significantly induced by salt stress. Ectopic expression of *UGT85A5* in tobacco enhanced the salt stress tolerance in the transgenic plants. There were higher seed germination rates, better plant growth and less chlorophyll loss in transgenic lines compared to wild type plants under salt stress. This enhanced tolerance of salt stress was correlated with increased accumulations of proline and soluble sugars, but with decreases in malondialdehyde accumulation and Na^+^/K^+^ ratio in *UGT85A5*-expressing tobacco. Furthermore, during salt stress, expression of several carbohydrate metabolism-related genes including those for sucrose synthase, sucrose-phosphate synthase, hexose transporter and a group2 LEA protein were obviously upregulated in *UGT85A5*-expressing transgenic plants compared with wild type controls. Thus, these findings suggest a specific protective role of this glycosyltransferase against salt stress and provide a genetic engineering strategy to improve salt tolerance of crops.

## Introduction

Salinity is a major abiotic stress that impacts on plant growth and productivity. Almost all aspects of plant activities are directly or indirectly affected by salt stress, which is generally defined as the presence of excessive amounts of soluble salts that hinders or affects the normal functions needed for plant growth [1]. Ionic and osmotic stresses are considered to be two main effects caused by salt stress on plants [2]. Previous studies indicated that salt stress-stimulated physiological imbalance increases the level of reactive oxygen species (ROS) in plant cells, leading to oxidative stress [3]–[5]. To cope with these environmental challenges, plants have evolved elaborate mechanisms to trigger stress responses at both molecular and morphological levels. Up to now, salt stress responses have been revealed to be mediated by many pathways. For instance, the SOS pathway genes *SOS1*, *SOS2* and *SOS3* play significant roles in resisting salt stress by regulating ionic homeostasis and Na^+^ tolerance [6]–[8]. A similar effect was also observed for the *AtNHX1* gene, which encodes a vacuolar Na^+^/H^+^ antiporter and contributes to the transportation of Na^+^ [9]. Mitogen-activated-protein kinase (MAPK) cascades are proposed to function in salt stress tolerance by regulating osmolyte biosynthesis [10], [11]. It is documented that overexpression of a tobacco ethylene receptor NTHK1 in Arabidopsis resulted in altered salinity sensitivity, indicating that ethylene signaling is also required in salt stress response [12]. In addition, a number of other genes such as late-embryogenesis-abundant (LEA) proteins, RD29A [13] and CBF/DREB transcription factor *ect*, which are strongly induced by salt stress, also act as critical regulators in mediating plant salt stress adaptation [14], [15].

Glycosylation occurs in a wide range of biological processes in plants. Glycosyltransferases (GTs) are enzymes responsible for glycosylation of plant compounds, which catalyze the transfer of sugar moieties from activated donor molecules to specific acceptor molecules, for example sugars, lipids, proteins, nucleic acids, antibiotics and other small molecules, with either retention or inversion of configuration at the anomeric center [16]. Until now, GTs have been classified into 94 distinct families based on their gene products or by sequence comparison with carbohydrate active enzymes (CAZy) (http://www.cazy.org/) [17], of which Family 1 is the largest and is closely related to plant functions. GTs of Family 1 characteristically contain a carboxy-terminal consensus sequence termed ‘plant secondary product glycosyltransferase box’ (PSPG box) and use uridine 5′-diphospho sugars as the sugar donors, and are thus named UDP-sugar glycosyltransferases (UGTs) [18]–[20].

Comprehensive analysis of the Arabidopsis genome revealed multigene GT families consisting of 119 putative UGT genes [21]. In plants, UGTs are generally localized in the cytosol, and are involved in the biosynthesis of plant natural products such as flavonoids, phenylpropanoids, terpenoids and steroids, and in the regulation of plant hormones [22]. It is now recognized that UGT action, by adding a sugar moiety to the acceptors, can change chemical properties and bioactivity of plant molecules and enable their access to membrane transporter systems [18], [19]. Therefore, UGTs might play important roles in maintaining cell homeostasis, regulating plant growth and improving their defense responses to stressful environments [18], [19]. For instance, several UGTs have been shown to glycosylate phytohormones *in vitro* and participate in phytohormone homeostasis *in vivo* [23]–[27]. Several publications indicate that knockout of *ugt73b3* and *ugt73b5*, or overexpression of *UGT74F2* and *UGT76B1*, significantly altered plant resistance to pathogen infection, implying novel roles of UGTs in plant defense responses [28]–[30]. In addition, UGTs have also been identified to participate in regulating plant adaptation to abiotic stresses. A recent study showed that overexpression of *UGT74E2*, encoding an indole-3-butyric acid (IBA) GT, greatly affected IBA homeostasis and the expression of various downstream genes, including transcription factors, glycosidases or genes involved in hormone signaling (e.g. *DIN2*, *P450*, *RD20*, *ORG1* and *PCC1*) leading to increased tolerance to salinity and drought stress in *Arabidopsis thaliana* [31]. *UGT73B2* loss-of-function endowed the mutant with oxidative stress tolerance [32]. Family 1 are the largest GT family that has various potential substrates from plant secondary metabolism. The widespread occurrence, diversity and complexity of the glycosides throughout the plant kingdom indicate that Family 1 GTs might have broad functionality. Although some UGTs have been chemically characterized, very little is known about their actual biological functions and molecular mechanisms, particularly during plant growth, development and response to external environments.

In this study, a novel Arabidopsis GT gene *UGT85A5*, a group G member of Family 1, was identified as involved in regulating plant salt stress tolerance. The expression of *UGT85A5* was strongly induced by NaCl. Ectopic expression of *UGT85A5* in tobacco resulted in enhanced salt tolerance in transgenic compared to wild type (WT) plants. The data presented here demonstrate, for the first time, that *UGT85A5* plays a significant role in enhancing plant salt stress tolerance.

## Results

### Induction of the Arabidopsis *UGT85A5* in response to salt stress

**Figure 1:**
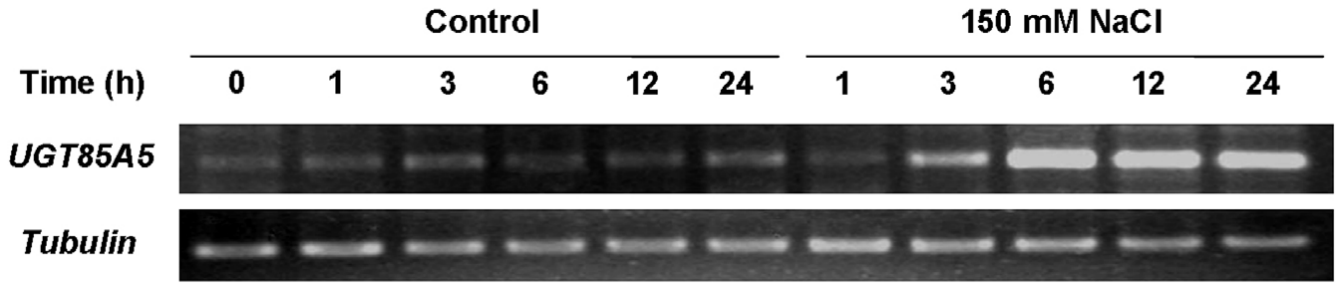
Expression patterns of *UGT85A5* in response to salt stress. Two-week-old Arabidopsis seedlings were treated with 150 mM NaCl as salt treatment and water as control. RT-PCR analysis was performed with *UGT85A5* specific primers using the RNA isolated from the Arabidopsis plants. The *Tubulin* was used as an internal control.

Two-week-old Arabidopsis seedlings were treated with 150 mM NaCl for different time courses to determine the expression pattern of *UGT85A5* in response to salt stress. RT-PCR analysis revealed that, compared with untreated controls, the transcript level of *UGT85A5* was remarkably upregulated under NaCl treatment (Figure 1). It was induced at 3 h, peaked at 6 h and remained at a very high level at 24 h. These results indicated that *UGT85A5* expression was salt inducible and that the gene was a direct or indirect target of a pathway that controlled salt-responsive gene expression.

### Generation of *UGT85A5* transgenic tobacco plants

**Figure 2:**
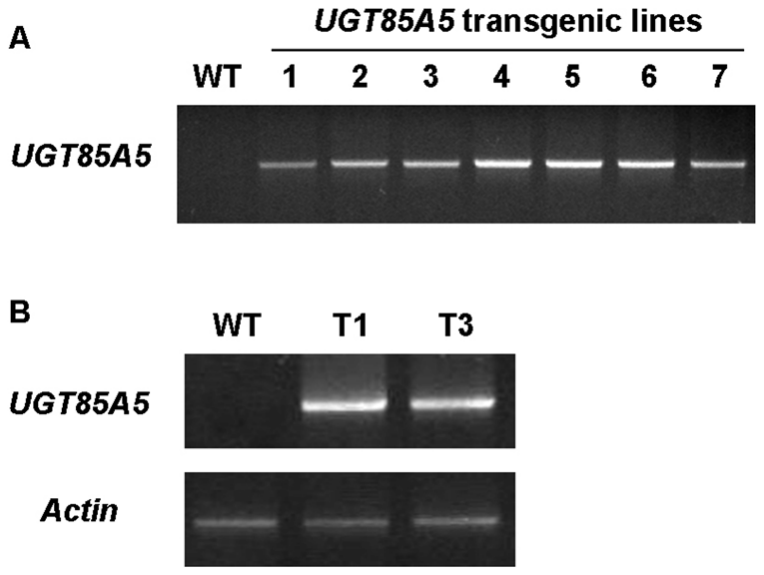
Identification of *UGT85A5* transgenic tobacco plants. (A) PCR analyses of T_0_ transgenic tobacco lines. (B) RT-PCR analyses of *UGT85A5* expression in homologous transgenic lines of T_2_ generation. The expression level of the *Actin* gene was used as an internal control. WT: wild type plants. T1 and T3: homologous transgenic tobacco lines of T_2_ generation.

To investigate the physiological function of *UGT85A5* in response to salt stress, transgenic tobacco that ectopically expressed *UGT85A5* was generated. A total of seven independent T_0_ transgenic lines were obtained by kanamycin selection and PCR identification. The expected *UGT85A5* fragment was amplified in the genomic DNA of transgenic lines but not in the non-transformed plants (Figure 2A). To assess the expression level of the *UGT85A5* gene in transgenic tobacco plants, 4-week-old homozygous transgenic seedlings of T_2_ generation were subjected to reverse transcription (RT)-PCR analysis. Of these, two lines (T_1_ and T_3_) showed the highest expression of *UGT85A5* and were chosen for further analysis (Figure 2B).

### Salt tolerance analyses of *UGT85A5* transgenic plants

To investigate whether constitutive expression of *UGT85A5* in tobacco improved salt stress tolerance, both WT and transgenic plants were challenged with NaCl treatment during both seed germination and vegetative stages.

**Figure 3:**
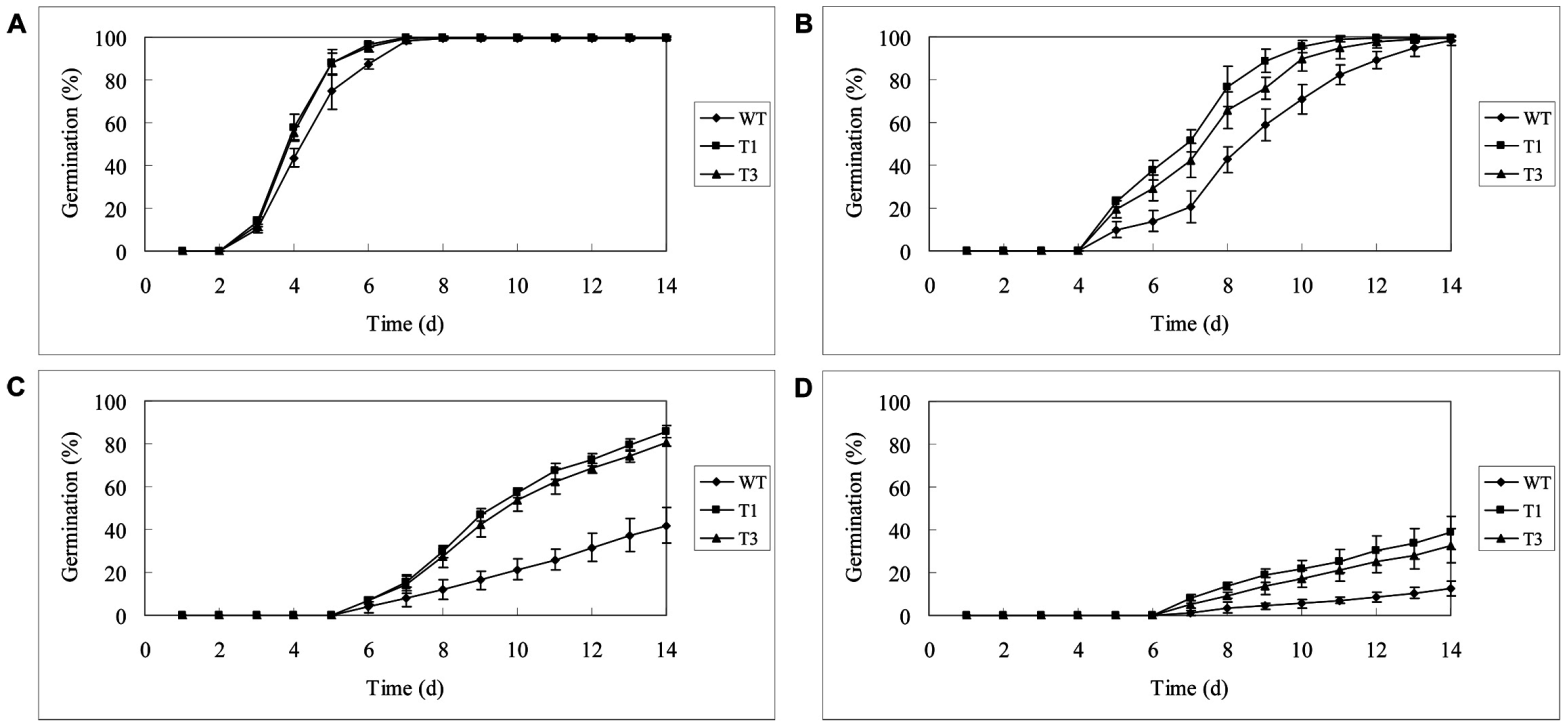
Seed germination under salt stress. Seeds of wild-type (WT) and *UGT85A5* transgenic lines (T1 and T3) were germinated on MS medium supplemented with different concentrations of NaCl. (A) Control; (B) 100 mM NaCl; (C) 150 mM NaCl and (D) 200 mM NaCl. The values are means of three independent replicates (each with 150 seeds).

Seeds of WT and transgenic lines (T_1_ and T_3_) were surface sterilized and germinated on Murashige and Skoog (MS) medium supplemented with 0, 100, 150 and 200 mM NaCl, respectively. Overexpression of *UGT85A5* in tobacco significantly promoted germination in untreated conditions (Figure 3A), suggesting that a regulatory network involved in seed germination influenced by UGT85A5 action was conserved in tobacco. In the presence of 100 mM NaCl, although there was no difference in the final germination rate of all seeds, those of T_1_ and T_3_ germinated faster than WT: >90% of T_1_ and T_3_ seeds germinated after 10 d, but only 70% for WT (Figure 3B). Additionally, under harsher salt stress conditions, the transgenic lines showed remarkably higher germination rate. About 80% transgenic seeds under 150 mM NaCl and 35% under 200 mM NaCl germinated after 14 d; however, only 40 and 10% of WT seeds germinated, respectively (Figure 3C and 3D).

**Figure 4:**
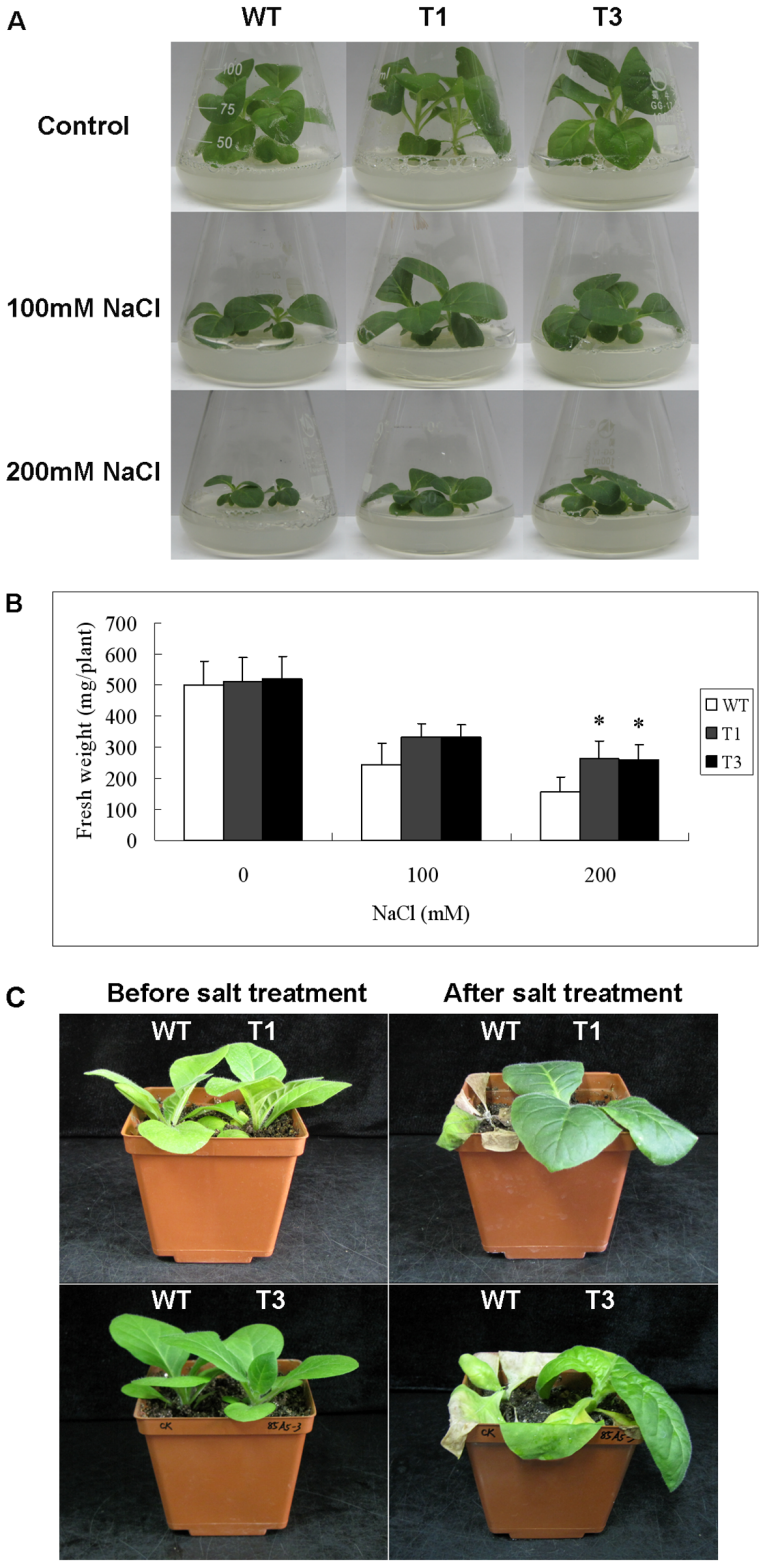
Effects of *UGT85A5* overexpression on salt stress tolerance. (A) Phenotype of WT and transgenic lines growing on MS medium supplemented with different concentrations of NaCl. (B) Fresh weight of WT and transgenic seedlings. (C) Phenotype of WT and transgenic plants watered with NaCl solution (from 100 mM to 300 mM) for 4 weeks.

We next examined whether overexpressing *UGT85A5* in tobacco influenced the growth of seedlings under salt treatment. Seeds of WT and transgenic plants were germinated and grown on MS medium for 3 weeks, and then the seedlings were transferred onto MS medium containing 0, 100 and 200 mM NaCl and grown for another 4 weeks. Both T_1_ and T_3_ transgenic plants grew better compared with WT (Figure 4A), and total fresh weight (mg plant^–1^) of the transgenic lines under salt stress conditions was significantly (*P*<0.05) higher than that of WT plants (Figure 4B).

Salt stress tolerance was also investigated for large plants growing in a greenhouse. Six-week-old WT and transgenic plants grown in the same pot were watered with NaCl solution (from 100 to 300 mM as described in Methods section) for 4 weeks. The WT seedlings showed severe chlorosis, stunted phenotypes and ultimately died, whereas the transgenic seedlings continued to grow (Figure 4C) – demonstrating that ectopic expression of *UGT85A5* greatly enhanced the salt stress tolerance of these transgenic plants.

### Leaf disk assay of *UGT85A5* transgenic tobacco plants

**Figure 5:**
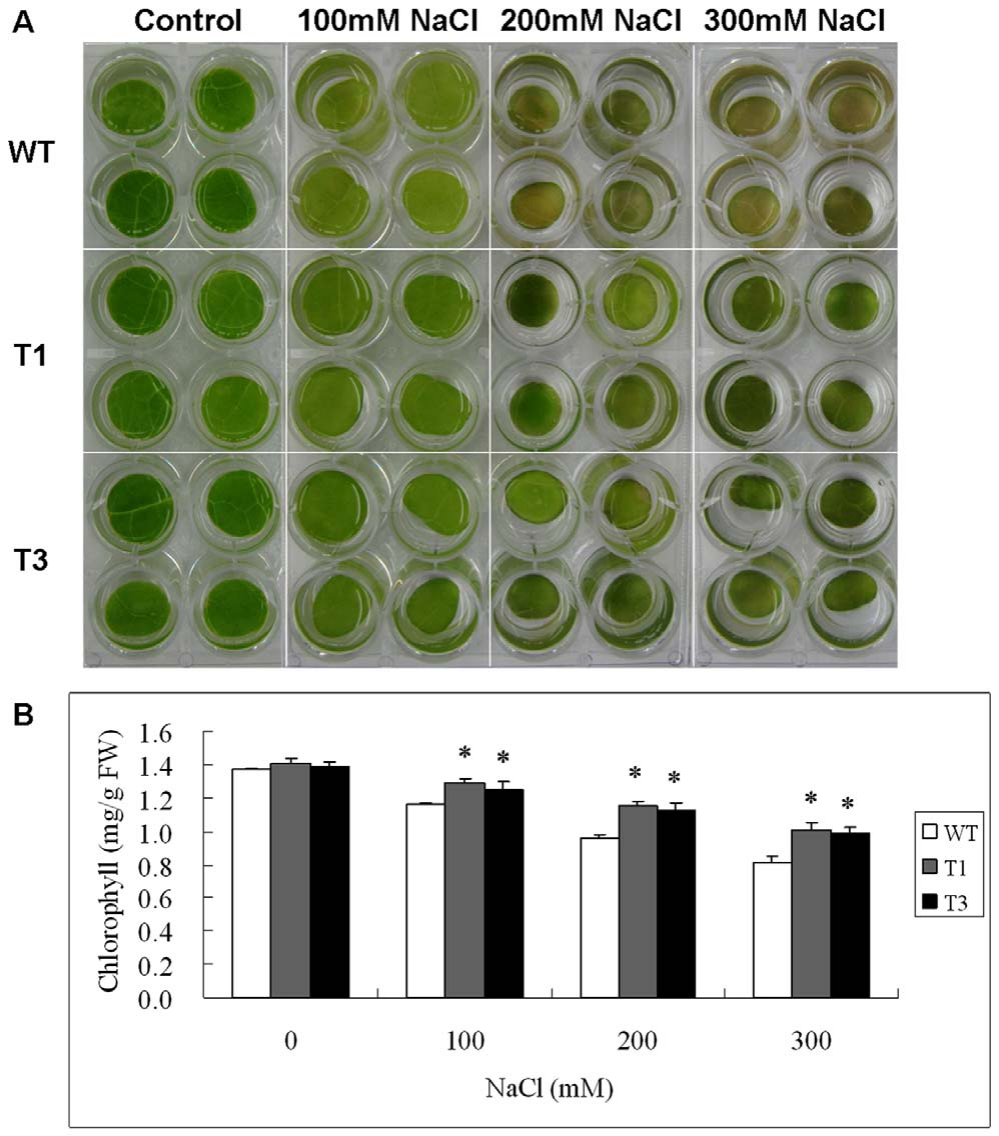
Evaluation of salt stress tolerance of the *UGT85A5* transgenic lines by leaf disc assay. (A) Representative picture that shows phenotype differences of leaf discs between WT and transgenic lines. (B) Chlorophyll contents of leaf discs from WT and transgenic plants treated with various concentrations of NaCl.

A leaf disk assay is a reliable index of the damage to the photosynthesis apparatus under stress. Leaf disks of WT and transgenic tobacco plants were subjected to water solutions containing 0, 100, 200 and 300 mM NaCl respectively to evaluate their tolerance to salt stress. After treatment for 3 d, a yellow phenotype was observed in WT leaf disks, whereas transgenic disks remained green (Figure 5A). Additionally, further chlorophyll measurement showed more dramatic chlorophyll loss in the WT leaf disks than the transgenic lines under salt stress. There was no difference in chlorophyll contents between WT and transgenic plants without treatment (Figure 5B). However, in the presence of 100 mM NaCl, there was 20% chlorophyll loss in WT leaf disks, but only 10% in transgenic ones – the discrepancy was greater with increased NaCl concentrations (Figure 5B). These results suggested that expression of *UGT85A5* in tobacco greatly enhanced its photosynthetic abilities under salt stress.

### Evaluation of soluble osmolyte content, lipid peroxidation and Na^+^/K^+^ ratio in *UGT85A5* transgenic plants

Different chemical compounds, such as proline, soluble sugars, sugar alcohols and quaternary ammonium compounds, are known to accumulate in response to abiotic stresses [33], [34]. Therefore, to gain further insight into the physiological relevance of *UGT85A5*, we analyzed changes of these biochemical markers in transgenic and WT plants under salt stress conditions.

**Figure 6:**
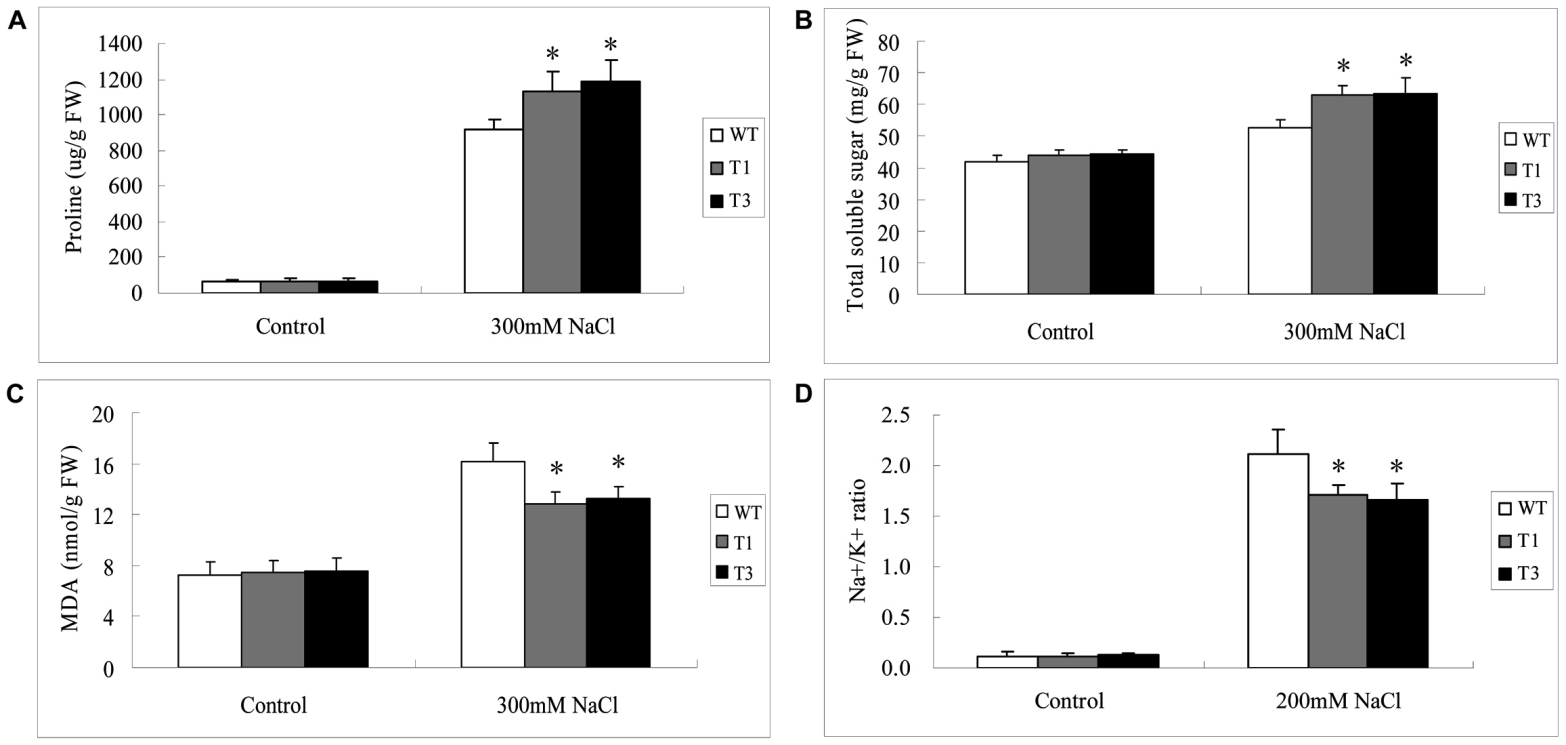
Changes in soluble osmolyte content, lipid peroxidation and Na^+^/K^+^ ratio of the WT and transgenic lines under salt stress. Leaves from plants treated with salt stress were collected and subjected to the determination. (A) Proline content; (B) Soluble sugars content; (C) MDA content; (D) Na^+^/K^+^ ratio.

Under normal conditions, a similar proline level was detected between WT and transgenic lines. Under salt stress, however, there were 17- and 16-fold higher proline levels in T_1_ and T_3_ transgenic plants, respectively. There was only a 12-fold increase of proline level in WT plants (Figure 6A).

A similar result was obtained in content of soluble sugars. There were equal soluble sugar levels in untreated WT and transgenic lines. After treatment with 300 mM NaCl, in contrast to a 20% increase in WT plants, the soluble sugar contents significantly increased by 40 and 38% in T_1_ and T_3_ plants, respectively (Figure 6B).

To measure lipid peroxidation, malondialdehyde (MDA) content was assessed in 300 mM NaCl stressed WT and transgenic plants – about 40% more MDA accumulated in WT than in transgenic lines (Figure 6C).

Moreover, the Na^+^/K^+^ ratio was also examined to see whether it was altered in *UGT85A5* transgenic plants. There were similar Na^+^/K^+^ ratios in both the transgenic and WT plants under normal conditions (Figure 6D). However, this ratio increased 18-fold in WT plants, in contrast to 12-fold in transgenic plants under salt stress conditions.

The significant level changes in the abovementioned components in the transgenic lines provided further evidence that ectopically expressing *UGT85A5* in tobacco greatly protected the plants from damage caused by salt stress, which in turn enhanced salt stress tolerance in the transgenic tobacco.

### Expression analysis of stress-related genes in *UGT85A5* transgenic plants

**Figure 7:**
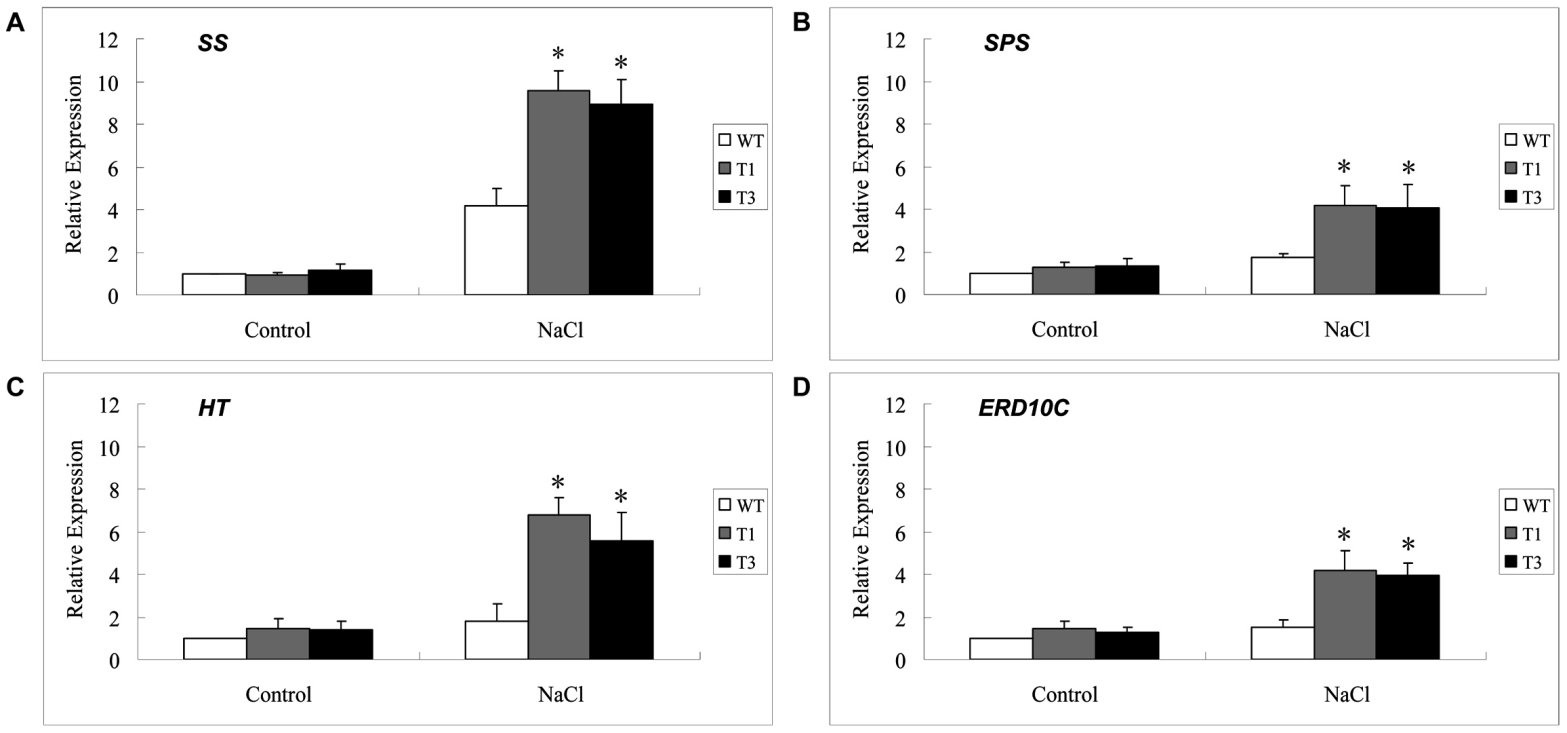
Expression analysis of stress-related genes in *UGT85A5* transgenic plants by qRT-PCR. The transcript levels of (A) *SS*, (B) *SPS*, (C) *HT* and (D) *ERD10C* in WT and transgenic tobacco plants were analyzed under both normal and salt treatment conditions.

To investigate how *UGT85A5* increased salt stress tolerance, the expression of some stress-related genes were analyzed. When treated with high salinity, the steady-state mRNA levels of carbohydrate metabolism-related genes encoding sucrose synthase (*SS*) [35], sucrose-phosphate synthase (*SPS*) [36], hexose transporter (*HT*) [37] and a group2 LEA protein (*ERD10C*) [38] in transgenic lines were upregulated 2- to 4-fold of that in WT plants (Figure 7). However, the transcript levels of these genes were similar between transgenic lines and WT plants under normal conditions. In summary, these results showed that increased salt resistance of *UGT85A5* overexpressing plants correlated with elevated expression levels of a subset of genes with known roles in stress responses.

## Discussion

Salt stress tolerance in plants is a complex trait involving multiple physiological and biochemical mechanisms. To endow the plants with greater flexibility and plasticity under stress conditions, it is essential to make dramatic modifications to a variety of small molecules, either intrinsic or extrinsic, including hormones, metabolites and xenobiotics [39], [40]. In particular, glycosylation catalyzed by a superfamily of GTs plays significant roles in modulating the solubility, stability, bioavailability and bioactivity of various small molecules [18], 19,41. Recent studies have demonstrated that GTs play important roles in modulating plant tolerance to abiotic stresses [31], [32]. In this study, for the first time, we demonstrated that ectopic expression of the Arabidopsis GT gene *UGT85A5* enhanced the adaptation of the transgenic tobacco plants under salt stress. A BLAST search at NCBI (http://www.ncbi.nlm.nih.gov/Blast) showed that *UGT85A5* has two homologs in tobacco: *NTGT5a* (GenBank AB176523.1) and *NTGT5b* (GenBank AB176524.1). They share 62% sequence identity to *UGT85A5* on a protein level. However, there is no report concerning the physiological role of *NTGT5a* and *NTGT5b*. *UGT85A5* is a gene of Arabidopsis, and since its expression is salt inducible and its ectopic expression can increase tobacco tolerance to salt stress, we speculate that *UGT85A5* may be involved in a regulation pathway of Arabidopsis in response to environmental stress. Our research on the effects of *UGT85A5* in Arabidopsis using its overexpresssors and mutants is now in progress. Tobacco is not only a crop plant but also material for easy genetic transformation – thus, we chose tobacco as the initial transgenic plants for the present study. Our data suggest that *UGT85A5* has promising utility in improving salinity tolerance in crops, at least in tobacco.

In the context of photosynthesis, chlorophyll is an essential factor as a driving force for plant growth and biomass production. The ability to maintain photosynthesis rate under environmental stresses is fundamental for the maintenance of plant growth and development [42]. The leaf disk assay in this study showed less chlorophyll loss in transgenic than in WT plants under salt stress conditions, indicating higher photosynthetic capacity in *UGT85A5* transgenic than in WT plants.

Organic osmolytes such as proline and soluble sugars work in a variety of ways, such as in protection of cellular structures, detoxification of enzymes and scavenging of ROS alone or in combination with other defense-related enzyme systems [43], [44]. These compounds also confer integrity to the membranes and keep the photosynthetic system functioning [44], which is in agreement with our data showing higher chlorophyll contents in transgenic than in WT plants. The plasmalemma is known to play critical roles in perceiving and transferring signals; however, its fluidity can easily be affected by abiotic stresses. Compatible solutes are essential in protecting the membranes from various damages [44] and can help to overcome the osmotic stress of salinity. Therefore, better growth of transgenic lines under salt stress might be partly attributed to increased levels of osmolytes. Additionally, *UGT85A5* transgenic plants exhibited less lipid peroxidation, as revealed by low MDA levels of these plants, demonstrating that the transgenic plants suffered less oxidative damage than WT plants under salt stress conditions. Ion measurements showed lower Na^+^/K^+^ ratios in leaves of *UGT85A5* transgenic compared to WT plants under salt stress, demonstrating that the transgenic plants suffered lower ion toxicity, which is a strong indicator of salt stress tolerance. UGT85A5 may influence ion accumulation or transportation by catalyzing the glycosylation of some molecules, leading to regulation of some ion transporters.

In the present study, the higher expression level of *SS*, *SPS* and *HT* in transgenic plants was consistent with the elevated osmolyte concentration under salt conditions, resulting in improved ability to adjust in response to salt stress. The protein encoded by *ERD10C* is thought to protect macromolecules and membranes under stress conditions [38], [45], which is consistent with our finding of higher expression of *ERD10C* in transgenic than WT plants under salt stress. All these results suggested that ectopic expression of *UGT85A5* positively altered the expression of these stress-related genes, leading to enhanced tolerance to salt stress in transgenic lines. UGT85A5 has previously been identified as being induced by a combination of drought and heat stress [46]. We analyzed the *UGT85A5* promoter and found that it has many binding sites of transcription factors responding to environment signals, such as ABRE binding site motif (ABF binding site), G-box promoter motif and the ACGT elements (Figure S1), which have been reported to be involved in responses to dehydration and ABA treatment [47]–[49]. Interestingly, the *UGT85A5* promoter has a short sequence (CACGTGGC) which contains three motifs in the same site, i.e. ABRE binding site motif, G-box promoter motif and ACGT element (Figure S1). The characteristics of the *UGT85A5* promoter suggest that *UGT85A5* may be a target of regulatory networks that control salt, drought, cold, light and ABA responsiveness.

In conclusion, our results demonstrated that *UGT85A5* was responsive to salt stress, and ectopic expression of *UGT85A5* strongly enhanced salt tolerance in transgenic plants. *UGT85A5* encodes a putative GT belonging to the group G of Family 1 UGTs [41]. However, almost nothing is really known about its enzyme activity and its substrates. *UGT85A1*, encoding a cytokinin GT, is the only gene of group G whose substrate has been identified so far [50]. However, UGT85A5 did not display any activity toward cytokinins in our research (unpublished data). Further investigations concerning UGT85A5 activity and its substrates might be helpful in understanding the stress-tolerance mechanism with which it is involved.

## Materials and Methods

### Plant materials and growth conditions

WT *Arabidopsis thaliana* Columbia ecotype (Col-0) and tobacco (*Nicotiana tabacum* Linn, Wisconsin 38) were used in this study. Seedlings of Arabidopsis plants and tobacco were grown on MS medium in a culture room at 23±2°C under a 14/10 h light/dark photoperiod, and a light intensity of 60 µmol m^–2^ s^–1^. Plants were grown in flowerpots in soil–perlite mixtures in a greenhouse at 24±1°C under a 16/8 h light/dark photoperiod and a light intensity of 100 µmol m^–2^ s^–1^.

### Vector construction and generation of transgenic plants

The full-length cDNA of the *UGT85A5* gene (AT1G22370) was amplified using the following primers (UGT85A5-F: 5′-GCGTCTAGAATGGCGTCTATGCTGTTC-3′ and UGT85A5-R: 5′-GCGGAGCTCCTACTCCCCTAAAAGAACCT-3′) and cloned into the pBI121 vector driven by the cauliflower mosaic virus (CaMV) 35 S promoter. The recombinant plasmid was transferred into *Agrobacterium* (strain LBA4404) for tobacco transformation using the leaf disk method [51], and the transformants were screened by kanamycin (100 mg l^–1^). The genomic DNA of WT and T_0_ tobacco plants was used to amplify the target gene *UGT85A5* by PCR using the primers mentioned above (i.e. UGT85A5-F and UGT85A5-R). For the RT-PCR analysis, total RNA of WT and transgenic tobacco plants (homozygous T_2_ generation) was extracted and reverse transcribed into cDNA. The resulting cDNA was used for the amplification of the target gene using gene-specific primers (KY-LP: 5′-GGCTTCTTGGCTTATCTACA-3′ and KY-RP: 5′-ACTCCTCAACCTCCTCCCTC-3′). The *Actin* gene was amplified using the following primers (ACT-F: 5′-ATGCCCTCCCACATGCTATTC-3′ and ACT-R: 5′-AACATGGTAGAGCCACCACTG-3′) as an internal reference.

### Salt stress assay in Arabidopsis and transgenic tobacco lines

Two-week-old Arabidopsis seedlings grown on MS solid medium were used to determine the expression pattern of *UGT85A5* under salt stress. Parts of the seedlings were treated with 150 mM NaCl, while other seedlings were treated with water as controls. Plants were sampled at 0, 1, 3, 6, 12 and 24 h after each treatment. Total RNA was extracted for RT-PCR analysis using the gene-specific primers (KY-LP and KY-RP). The *Tubulin* gene was amplified using the following primers (TUB-F: 5′-CTCAAGAGGTTCTCAGCAGTA-3′ and TUB-R: 5′-TCACCTTCTTCATCCGCAGTT-3′) as an internal reference.

To determine the effects of salt stress on seed germination, seeds of WT and transgenic tobacco plants (homozygous T_2_ generation used for all experiments in this study) were surface sterilized and sown on MS solid medium supplemented with different concentrations of NaCl (0, 100, 150 and 200 mM). The plates were placed under greenhouse conditions for 14 d. The number of germinated seeds was counted daily after sowing.

For evaluation of salt tolerance at seedling stage, 3-week-old seedlings of the WT and transgenic tobacco lines grown on MS medium were transferred to MS medium supplemented with various concentrations of NaCl (0, 100 and 200 mM) and were allowed to grow for 4 weeks. The seedling growth was observed and the fresh weight determined (mg·plant^–1^).

To evaluate salinity tolerance under greenhouse conditions, 1-month-old seedlings of WT and transgenic plants grown on MS medium were transferred to pots containing a mixture of garden soil and sand (3 1) and grown in a greenhouse for two more weeks before exposure to salt stress treatments. Then each line was irrigated with NaCl solution every 3 d. The NaCl concentration used initially was 100 mM and then stepped up in 50-mM increments until the final concentration of 300 mM was achieved. The phenotype of plants was photographed after 30 d of salt treatment.

For the leaf disk floating assay, leaf disks with a diameter of 1 cm (five disks per treatment) were prepared from leaves of identical development stage of both WT and transgenic plants and floated on solutions of different NaCl concentrations (0, 100, 200 and 300 mM) for 72 h. The phenotype of leaf disks was photographed and the total chlorophyll content in each sample was measured after salt treatment. The chlorophyll content [Chls a+b] was calculated after extraction in aqueous 80% acetone using the following formulae (in g·l^–1^): [Chls a+b] = (20.21 × A645)+(8.02 × A663) [52]. A663 and A645 represent absorbance values read at 663 and 645 nm wavelengths, respectively.

### Determination of proline, soluble sugar, MDA and ion content

Three-week-old seedlings of WT and transgenic lines grown on MS medium were transferred to soil for additional growth duration of 3 weeks. Selected WT and transgenic plants of the same age and size were either transferred to pots to impose salt stress (300 mM NaCl solution) on them or to pots without salt treatment as experimental controls. After 7 d of treatment, leaves were collected for physiological assays. Proline accumulation in tobacco plants following salt treatment was measured according to the method of Shan et al. [53]. Soluble sugars were essentially extracted using the procedure of Li et al. [54]. MDA was assayed for indirect evaluation of lipid peroxidation using thiobarbituric acid, as described by Hodges et al. [55].

To determine the ion contents, 3-week-old seedlings of the WT and transgenic lines were transferred to MS medium containing different concentration of NaCl (0 and 200 mM) and were allowed to grow for 1 month. Then leaves from WT and transgenic plants were collected and dried at 80°C for 48 h. The dried materials were extracted with 1 N HNO_3_ as described by Storey [56]. The Na^+^ and K^+^ contents were measured in the filtrate using atomic absorption spectrophotometry.

### Total RNA extraction and qRT-PCR analysis

To study the expression level of stress-related genes, 4-week-old seedlings were treated with 300 mM NaCl for 6 h as the salt treatment, while seedlings treated with water were the controls. Total RNA was extracted using Trizol reagent (TaKaRa) and treated with RNase-free DNase I (TaKaRa). Reverse transcription reactions were performed for 15 min at 37°C using the PrimeScript RT reagent kit (TaKaRa). The resulting cDNA was used for qRT-PCR analysis. The qRT-PCR was performed using the SYBR Premix Ex Taq II kit (TaKaRa) according to the manufacturer's instructions and run on LightCycler480 (Roche) real-time PCR systems. Thermocycling conditions were 95°C for 30 s followed by 40 cycles of 95°C for 5 s, 60°C for 20 s, with a melting curve detected at 95°C for 15 s and 65°C for 15 s. The relative transcript level was normalized with *qActin* gene. All primers used in qRT-PCR were as follows: (1) qACT-F: 5′-GCAGGCATTCACGAAACGACTTA-3′ and qACT-R: 5′-AGCCAAAATAGAACCTCCAATCCAA-3′; (2) SS-F: 5′-TGGTTGGAGGAGACCGAAGG-3′ and SS-R: 5′-GAGTTCGCCATTCCTCACGC-3′; (3) SPS-F: 5′-GAATTCAGGCGCTTCGTTGTCA-3′ and SPS-R: 5′-ACCCCTAGTTTCTCCAGTGA-3′; (4) HT-F: 5′-TTGCCCGAGACGAAGAATGT-3′ and HT-R: 5′-GCGCAGAAGGATCAAATCCA-3′; and (5) ERD10C-F: 5′-GGAAGAAGAGAAGGCGGGTGA-3′ and ERD10C-R: 5′-GGTCTTTGAGTGATATCCTGGTA-3′.

### Statistical analysis

Experiments were repeated at least three times. *P*-values were determined by Student's *t*-test (the quantification of stress-related genes expression) or by one-way ANOVA using the protected least-significant difference (LSD) tests (the quantification of phenotypic differences). Data are expressed as mean values ± SE.

## Supporting Information

Figure S1 **The structure of the promoter region of the** ***UGT85A5*** **gene.** Promoter nucleotide numbers begin at the 5′ end of the *UGT85A5* mRNA (see gene annotation in TAIR). Sequences homologous to ABRE binding site motif (C/TACGTGGC), G-box promoter motif (CACGTG), GATA promoter motif (A/TGATAG/A), ACGT element (ACGT) are underlined. Note that some GATA promoter motifs are presented on the complementary strand.
(TIF)
