# Supplementary figures and images for: Ectopic Expression of Arabidopsis Glycosyltransferase UGT85A5 Enhances Salt Stress Tolerance in Tobacco
Source: PLoS One. 2013 Mar 22;8(3):e59924. doi: 10.1371/journal.pone.0059924 (PMC3606239; doi:10.1371/journal.pone.0059924)

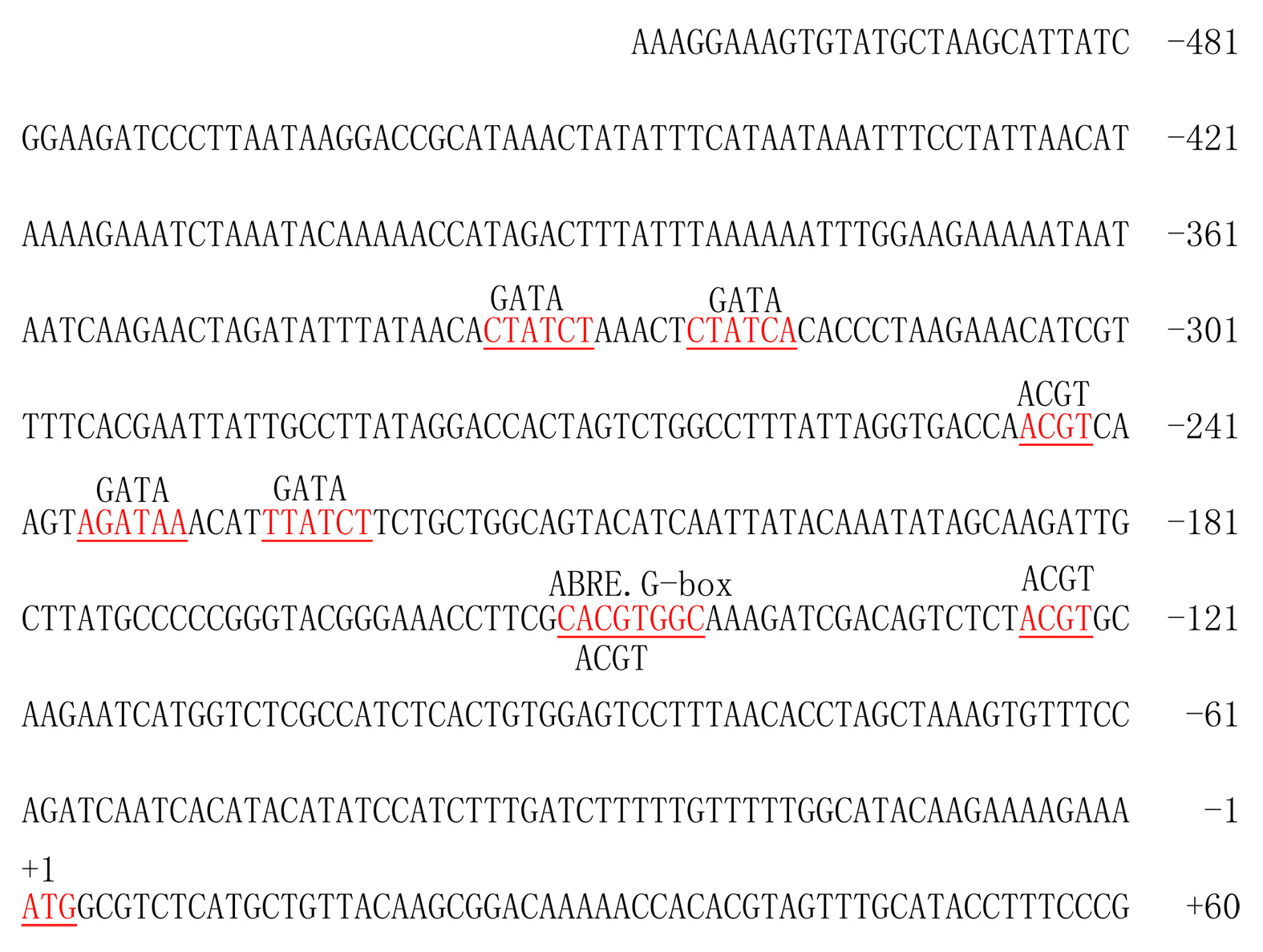

Supplement: Figure S1 — The structure of the promoter region of the UGT85A5 gene. Promoter nucleotide numbers begin at the 5′ end of the UGT85A5 mRNA (see gene annotation in TAIR). Sequences homologous to ABRE binding site motif (C/TACGTGGC), G-box promoter motif (CACGTG), GATA promoter motif (A/TGATAG/A), ACGT element (ACGT) are underlined. Note that some GATA promoter motifs are presented on the complementary strand. (TIF) [file pone.0059924.s001.tif]
